# Navigating Dravet syndrome in Spain: A cross‐sectional study of diagnosis, management, and care coordination

**DOI:** 10.1002/epi4.13012

**Published:** 2024-07-10

**Authors:** Sandra Solaz, Elena Cardenal‐Muñoz, Alicia Muñoz, Simona Giorgi, Federico V. Pallardó, Carlos Romá‐Mateo, José Ángel Aibar

**Affiliations:** ^1^ Departamento de Salud Hospital La Fe Centro de Salud de Silla Valencia Spain; ^2^ Dravet Syndrome Foundation Spain Madrid Spain; ^3^ Department of Physiology, Faculty of Medicine and Dentistry Universitat de València (UV) Valencia Spain; ^4^ Center for Biomedical Network Research on Rare Diseases (CIBERER) Institute of Health Carlos III Valencia Spain; ^5^ INCLIVA Biomedical Research Institute Valencia Spain

**Keywords:** diagnosis delay, Dravet syndrome, patient satisfaction, primary care, public healthcare resources

## Abstract

**Objectives:**

Dravet syndrome (DS) is a rare form of refractory epilepsy that begins in the first year of life. Approximately 85% of patients have a mutation in the SCN1A gene, which encodes a voltage‐gated sodium channel. The main objective of the present work was to assess the degree of knowledge of DS among Spanish primary care (PC) professionals, the communication flow between them and the pediatric neurologists (PNs), and the services available and resources offered to patients in Spain when searching for a diagnosis and adequate treatment.

**Methods:**

Two anonymized online surveys on DS diagnosis and patient management in PC were conducted with Spanish PC pediatricians (PCPs) and caregivers of DS patients in Spain.

**Results:**

Most PCPs are aware of genetic epilepsy but lack full knowledge of DS and patient advocacy groups (PAGs). Access to epilepsy treatments varies among regions, with many referrals to hospitals and pediatric neurologists. Diagnosis is often delayed, with misdiagnoses and frequent emergency room (ER) visits. Treatment involves multiple drugs, and sodium channel blockers are used, which are contraindicated in DS treatment. Improved training, resources, and communication are needed for early diagnosis.

**Significance:**

To improve the care and treatment of DS patients in Spain, early diagnosis is required and, possibly, specific efforts aimed at identifying patients in adulthood, generating socio‐sanitary structures that integrate social and health services to provide comprehensive care, taking into account the different features and comorbidities of the disease.

**Plain Language Summary:**

Dravet syndrome (DS) is a form of genetic epilepsy that starts within the first year of life. We present a study showing that, while family doctors are aware of genetic epilepsies, many don't have a complete understanding of DS. Unfortunately, getting the right diagnosis can take a long time, leading to unnecessary visits to the emergency room. Patients often need several medications, and sometimes they're given drugs that aren't recommended for DS. The takeaway is that training for doctors, more resources, and improved communication could help creating better healthcare systems and therefore give easier access to the right therapies.

**Key points**

- Spanish PCPs are generally aware of genetic epilepsy but lack full knowledge of DS and PAGs.
- DS diagnosis is often delayed, with misdiagnoses and frequent emergency room (ER) visits, especially in adult patients.
- Treatment involves multiple drugs, and sodium channel blockers are used, which are contraindicated in DS treatment.
- Training, resources, and communication between PCPs and PNs are needed for early diagnosis and better management of the disease.

## 1 INTRODUCTION

Dravet syndrome (DS; ORPHA33069) is a developmental and epileptic encephalopathy that begins between 4 and 12 months of age. The clinical course is the occurrence of generalized tonic–clonic or focal clonic seizures, sometimes of febrile origin. They are usually prolonged and resistant to antiseizure medications (ASMs), sometimes leading to status epilepticus (SE). The results of electroencephalography (EEG) and neuroimaging in this first phase are usually nonspecific and normal. Later in life, patients develop motor and language impairments and behavioral problems, as well as other comorbidities such as sleep disturbances, growth difficulties, and respiratory tract infections. With a mortality rate of 15%–20%, half of the DS premature deaths are caused by sudden unexpected death in epilepsy (SUDEP) (reviewed in ref. [1]).

Up to 85% of DS patients carry a pathogenic variant in the SCN1A gene, encoding the alpha subunit of the voltage‐gated sodium channel Na_v_1.1.^2^ Experts recommend requesting a genetic test for all children with prolonged seizures that are under 6 months of age with normal EEG and MRI.^3^, ^4^ There is a significant lack of awareness about the disease among healthcare professionals (HCPs)—mainly due to the low prevalence of the condition—and diagnostic delays are still significant although less frequent in younger patients.^5^, ^6^ This, together with the medical features of the disease, bears long‐term consequences on the quality of life (QoL) of patients and their families.^7^ In this context, the significance of multidisciplinary disease management is substantial. It encompasses not only healthcare services but also psychological, social, and educational support. These diverse components present considerable challenges for effective care coordination.^8^

Some studies propose that seizures are associated with worse cognitive development, while others claim that seizure burden is associated with increased comorbidities and decreased QoL.^9^ Hence, getting an early diagnosis is pivotal to prevent seizures, which have a positive impact on the evolution of the disease.^10^, ^11^ In addition, a fluid communication between primary care pediatricians (PCPs) and pediatric neurology services (PNSs) is essential. It improves the deterioration of the psychomotor development, the resistance to ASMs, and the use of contraindicated medications such as sodium channel blockers.^12^ Facilitating access of PCPs to specific training and establishing guidelines for referral to PNSs^10^ is of vital importance to achieve an adequate care for these patients. Pediatric guidelines recommend referral to a PN of a patient suffering from seizures if (i) there is SE, (ii) the seizure is focal, (iii) it is recurrent, (iv) the seizure is atypical or complex, (v) there is postictal neurological deficit, (vi) the seizure occurs before the 3 months of age, and/or (vii) epilepsy is suspected.^13^, ^14^, ^15^, ^16^ PAGs play a key role in early care, providing PC and PNSs with information related to the disease specificities and facilitating the interaction between them and the patients and caregivers.^17^

There exists scarce literature offering a global view on the interactions between PC staff, hospital specialists, and patients or on the availability for PCPs of specific resources for DS diagnosis and treatment in Spain. The present study assessed the degree of knowledge of DS among Spanish PC professionals, the communication flow between them and PNs, the natural progression of DS, diagnosis and referral times to PNs, and the impact on patient QoL.

## 2 METHODS

### 2.1 Design

The study was performed following the Helsinki Declaration, and it was approved by the Ethics Committee for Research with Medicinal Products of the Hospital Universitario and Politécnico La Fe in Valencia, Spain.

A comprehensive literature review was conducted to explore several aspects related to DS. These aspects included communication among HCPs, the availability of resources for DS diagnosis and treatment, the level of knowledge about DS among Spanish PCPs, diagnosis and referral timelines, and PCPs’ familiarity with PAGs. Following the review, two anonymous cross‐sectional surveys were developed through an iterative feedback process involving the authors.

The surveys were conducted online for 8 weeks between July and September 2021, one for Spanish PCPs from the National Health System (NHS) with more than 2 years of professional experience and another for adult caregivers of DS patients of any age residing in Spain. Participants were recruited through the Dravet Syndrome Foundation Spain (FSD) and invited to respond to the surveys via email or the FSD's social media channels (WhatsApp, Facebook, Twitter, Instagram, and LinkedIn). Efforts were made to ensure that both PCPs and caregivers were spread in the Spanish territory. Participants were provided with the study information sheet and the informed consent, which was electronically signed before participation in the study. Personal data were dissociated from the results in compliance with the EU General Data Protection Regulation (GDPR).

The survey of PCPs was divided into three blocks: (i) demographic data, (ii) clinical practice, and (iii) degree of knowledge about aspects of interest. The survey of DS caregivers was divided into four blocks: (i) demographic data, (ii) clinical data, (iii) treatment data, and (iv) severity of recent seizures and comorbidities. Response options were multiple‐choice, single‐choice, or free text. Only complete surveys were analyzed.

### 2.2 Statistical analyses

Variables were described using summary statistics: frequencies and percentages for categorical variables, and frequencies, mean, and range for continuous variables. The correlation between variables was calculated by regression analysis and *R* ^2^. Comparisons between categorical variables were made using the chi‐square test, performed with GraphPad Prism v8.0.1. All graphs, as well as correlation analyses, were made using Microsoft Excel.

## 3 RESULTS

### 3.1 Primary care pediatrician responses

The survey to PCPs was answered by 61 participants from various provinces in Spain (Table S1). PCPs were 33–66 years of age (mean 50.2 years), with a mean professional experience in pediatrics of 23.4 years (Table S2).

**FIGURE 1:**
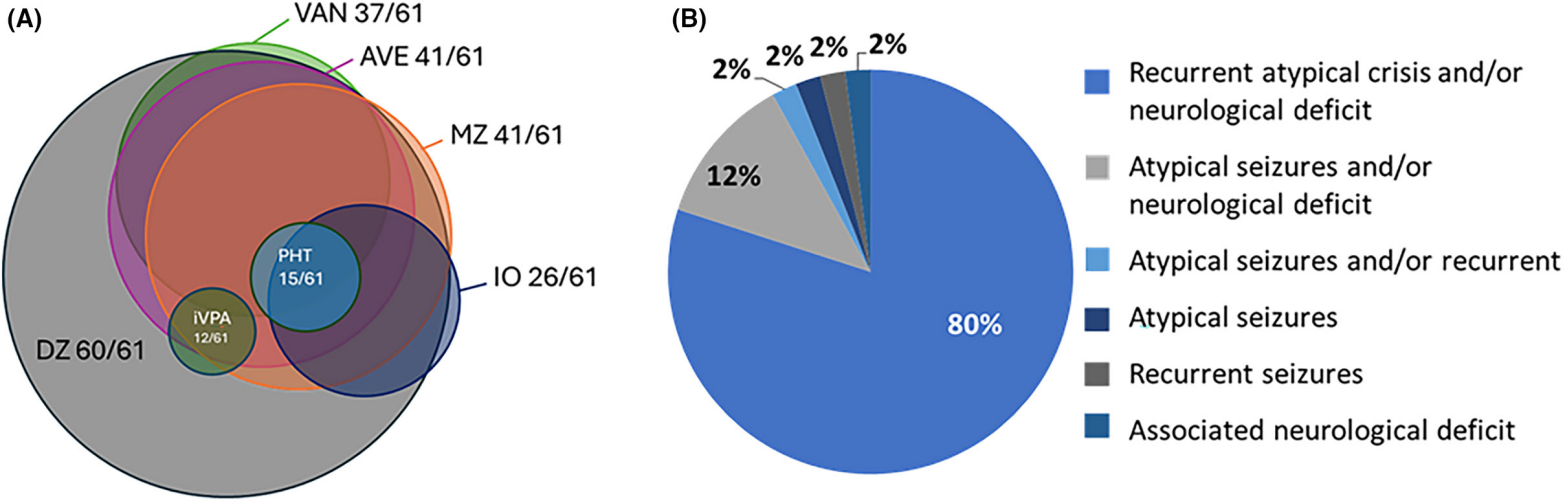
Tools and treatments for prolonged seizures available and referral criteria in PC. (A) Number of primary care pediatricians reporting access to corresponding treatments at their healthcare facility. Legend: Diazepam (DZ), Midazolam (MZ), Age‐appropriate intravenous access (AVE), Intraosseous (IO), Vascular Access Nurse (VAN), Intravenous Valproate (iVPA), Intravenous Phenytoin (PHT). (B) Percentage of referral criteria to pediatric neurology services in patients with febrile seizures under 1 year.

The questionnaire explored the presence of resources within the participants’ healthcare centers for managing prolonged seizures or SE, which is a common occurrence in DS patients as well as in other severe epilepsies.^18^ While the inquiry broadly pertains to the clinicians’ expertise in handling such events across various conditions, it also underscores the particular significance of these resources for the subset of patients with DS under their care. As a result, 98% (60/61) of respondents had diazepam (DZ) at their disposal, 67% (41/61) also had midazolam (MZ), 67% (41/61) had age‐appropriate peripheral venous access (AVE) and, to a lesser extent, nursing staff specialized in venous access in children (VAN, Vascular Access Nurse) (37/61) (Figure 1A). Other fundamental tools for the management of SE, like intraosseous accesses (IO) (26/61) or intravenous valproate (iVPA) (12/61) were not highly prevalent in healthcare centers. None of the respondents had at their disposal all the treatments recommended by the guidelines for the treatment of SE.^13^, ^14^, ^15^ When asked about the referral to PNS of children under 1 year of age after their first seizure, 80% (49/61) of PCPs referred patients in the event of atypical, recurrent, and/or neurological disorder‐associated seizures, while 12% (8/61) referred in the event of atypical seizures and/or neurological alteration (Figure 1B).

**FIGURE 2:**
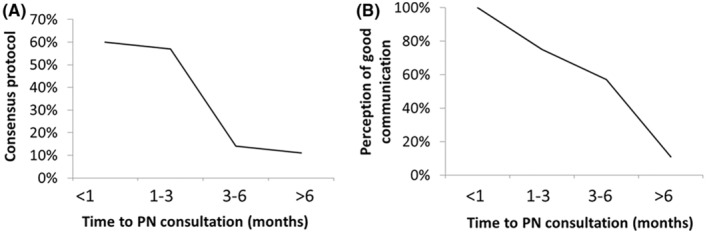
Waiting time for a pediatric neurologist (PN) consultation. (A) Relationship between waiting time for a PN consultation and availability of consensus protocols (*R* ^2^ = 0.85) or (B) Relationship between waiting time for PN consultation and the perception of good primary care (PC)‐NP communication (*R* ^2^ = 0.96). The figure illustrates the correlation between the perceived waiting time for patients to be seen at PN in relation to consensus protocols (%) and the perception of good communication between PC and PN (%).

Only 31% (19/61) of the participants reported the existence in their centers of agreed PC‐to‐PNs referral protocols. However, the communication between PCPs and PNs was well perceived by most of the PCPs surveyed (66%, 40/61). The reported delay in referring patients with suspected complex seizure varies, with 46% experiencing a delay of 1–3 months, followed by a 23% experiencing a delay of 3–6 months. Only 16% of surveyed PCPs reported a referral delay from PC to PNSs of less than 1 month, while 15% reported a delay of more than 6 months. Interestingly, when crossing the PCPs responses on the referral delays and the existence of referral consensus protocols or their perception of the PC and PNSs communication, the pediatricians who indicated referral delay times of less than 1 month confirmed the existence of agreed protocols (60%), compared to the 11% with no agreed protocols and delay times of more than 6 months (Figure 2A). A similar correlation is observed when analyzing the perception of communication between PC and PNSs: 100% of the pediatricians who indicated referral delay times of less than 1 month perceived the PC and PNSs communication as good, while those manifesting a delay greater than 6 months perceived a worse communication (Figure 2B).

Regarding the degree of knowledge among the PCPs of the epilepsies of genetic origin, 97% (59/61) claimed to know them. In the case of DS, 79% (48/61) of respondents were aware of the condition, while 21% (13/61) of PCPs was not.

In relation to the capacities of PAGs in patient and healthcare professional support, only 9 pediatricians (15%) were aware of the possibilities offered by these organizations—for example, psychosocial support, financial support, educational resources, etcetera. Nevertheless, 97% (59/61) of the PCPs stated that they recommended their patients to join a PAG after the diagnosis of a potentially serious illness, even though 60% of these professionals do not usually contact these organizations on a regular basis.

### 3.2 Caregiver responses

One hundred twenty‐two caregivers of patients with DS from various provinces in Spain participated in this study (Table S3); the majority of them (71% [87/122]) were mothers. The caregivers were between 28 and 66 years old (mean 43.8 years). Regarding the composition of the family unit, 107/122 DS patients (88%) lived with both parents, and 15/122 (12%) with a single parent. Eighty‐seven patients (71%) who lived with one or two parents also lived with siblings (Table S4). The age of the patients included in the study sample was between 8 months and 40 years old (mean age of 12.1 years), with a gender distribution of 56 women (46%) and 66 men (54%) (Table S4).

**FIGURE 3:**
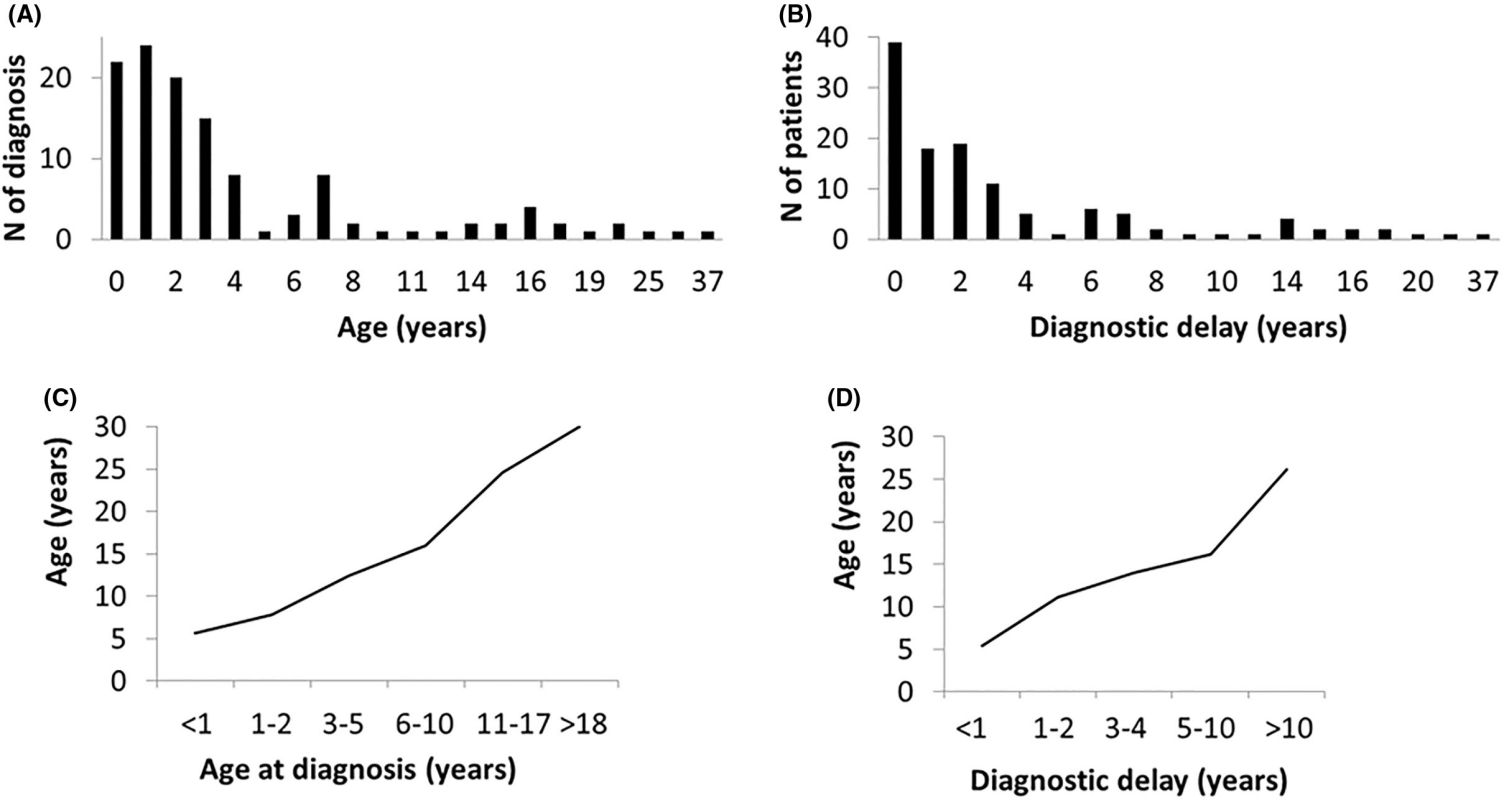
Patient age at diagnosis and diagnostic delay. (A) Patient age at diagnosis. The figure represents the age at which patients received a DS diagnosis. (B) Diagnostic delay, referred as to the time between the first seizure and the diagnosis. The figure represents the number of patients in our sample by years of diagnostic delay. (C) Relationships between age at diagnosis and current age of the patients (*R* ^2^ = 0.96). (D) Relationship between diagnostic delay time and current age of the patients (*R* ^2^ = 0.93).

Caregivers were then asked questions about the specific features of DS. The age of onset of the first seizure was between 0 and 13 months, with a mean age of 5 months. The most frequent age range for the onset of the disease was between 3 and 6 months (77%). The first seizure was mainly generalized tonic–clonic (68%), followed from far by focal (18%) and myoclonic (10%). This first seizure was febrile in 64% (78/122) of the respondents. Some factors triggering the first seizure were fever (50% [62/122]), immunization (38% [46/122]) (with fever [17/46] or without it [29/46]), infections (5% [6/122]), excitement (2.5% [3/122]) and/or heat (2.5% [3/122]). Almost half of the patients suffered more than 10 seizures during the first year of life; specifically, 24% (29/122) had between 10 and 20 seizures, while 27% (33/122) of patients had more than 20. In addition, 75% (92/122) of patients in the study sample presented SE in the first year of life. Among those, 58% presented between 1 and 4 SE episodes. The first EEG performed during the diagnostic process was mostly normal or nonspecific (76% [93/122] of the respondents), while in 10 patients (8%) the result of the first EEG was pathological, and 16% (19/122) was not sure of the result. Throughout the evolution of the disease, more than half of the patients (53%) maintained a normal EEG, with some individuals not showing any pathological EEG signs for as long as 39.7 years after the disease onset. The mean age of diagnosis of DS in the study sample was 6 years, ranging from less than 1 year to 37 years (Figure 3A).

Fifty‐four percent of the patients were diagnosed before the age of 2 years; 95% of them were under the age of 5 at the time of the survey. In contrast, 68% of the adult patients (≥18 years old) were diagnosed after the age of 10, while the rest of them (32% [8/25]) were diagnosed over the age of 18 (Figure 3A). As a result, the mean diagnostic delay―understood as the time between the first seizure and the mean age at diagnosis―was 5.6 years (Figure 3B). Sixty‐two percent of patients suffered a diagnostic delay of less than 2 years from the first seizure, with half of them (38/122) being diagnosed before 1 year from the first seizure. The current mean age of patients who waited less than a year for diagnosis is 5.4 years, and those who waited between 1 and 2 years for diagnosis were aged 11.1 years. Strikingly, 13% of patients, with a mean age of 26.2 years, received the DS diagnosis more than 10 years after the onset of the disease. As shown in Figure 3C,D, older patients received their diagnosis later in life, and they seem to have suffered from a more prolonged diagnostic delay.

**FIGURE 4:**
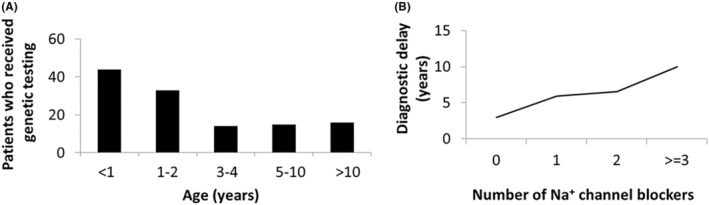
Genetic testing and treatment with sodium channel blockers. (A) Request for genetic testing by age groups. The figure represents the number of patients for whom genetic testing was requested, grouped by the age range at which the genetic test was requested. (B) Relationship between the number of sodium channel blockers prescribed to patients throughout their lives and diagnostic delay. The figure represents the diagnostic delay time in years in relation to the number of sodium channel blockers taken by the patients (*R* ^2^ = 0.94).

Genetic testing was requested in the first year of life for 50.8% of patients in the sample study. The average age at which a genetic test was requested for the patient was 4 years old. For more than half of the patients, the genetic test was requested before the age of 2 years, while for 13% of the patients, it was requested at more than 10 years of age, being the average age of this subgroup of 19 years (Figure 4A).

Besides diagnostic delays, misdiagnosis is another issue that may severely affect the management of DS patients.^19^ In this study sample, 70% (86/122) of patients received an initial diagnosis other than DS. The most common alternative diagnoses included febrile seizures or febrile seizures plus (31.8.9%, 27/85), a general diagnosis of epilepsy (25.9%, 22/85), neonatal epilepsy (14.1%, 12/85), or focal seizures (8.2%, 7/85). Less common diagnoses included atypical febrile seizures, metabolic deficits, or autism among others. The mean age at the time of the study of patients initially misdiagnosed was 13.7 years. From the remaining 36 patients (30%), 20 (17%) received a DS diagnosis after the genetic test results, while only 16 (13%) received an initial DS or DS‐like diagnosis. Seventeen caregivers (14%) reported their family to have been the one suggesting the DS diagnosis to their physician. Before receiving the diagnosis of DS, 47 patients (38%) had more than 20 visits to the ER. At the time of diagnosis, 32% (39/122) of patients received information about or were recommended to contact a PAG. At the time of conducting the survey, all families belonged to a PAG, namely Dravet Syndrome Foundation Spain.

Our data show that the most common antiseizure drug in DS patients was VPA (90%, 110/122), in the vast majority of them (97%, 108/110) in association with other drugs. The most frequent drug combinations were VPA and clobazam (64/122), VPA and stiripentol (49/122), and VPA, clobazam, and stiripentol (39/122). Seventy‐seven percent of the patients in the study sample were receiving treatment with 3 or more antiseizure medications at the time of the study. Thirty‐eight percent (46/122) of patients were being treated with new drugs specifically indicated for DS: 25% with fenfluramine and 13% with cannabidiol.

Importantly, 48% of the respondents (59/122) were treated at some point with sodium channel blockers, which are normally contraindicated for patients with DS.^5^ Interestingly, patients treated with sodium channel blockers suffered a longer mean diagnostic delay from the onset of symptoms than those not treated with sodium channel blockers (7.17 years vs. 2.95 years) (Figure 4B). They also had a higher mean age at the time of the study (15.9 years vs. 8.6 years).

Only 18% of patients who received a diagnosis within the year after the onset of symptoms were treated with sodium channel blockers. On the contrary, among the patients for whom the diagnosis was delayed between one and 2 years after the disease onset, the exposition to these contraindicated drugs increased to 45%, reaching 93% in patients with a diagnostic delay greater than 10 years. Fifty‐nine percent of the respondents (72/122) had a personalized emergency protocol, with instructions for the management of prolonged seizures, signed by their physician. Regarding treatments other than antiseizure medications, 65% (83/122) of the study cohort did not receive any additional treatment. The remaining 43 (35%) were being treated with vitamin supplements or amino acids (22/122), ketogenic diet (7/122), vagus nerve stimulation (4/122), treatment for attention deficit hyperactivity disorder (ADHD, 8/122), antipsychotic treatment (5/122), and nutrition therapy other than ketogenic diet (4/122).

**FIGURE 5:**
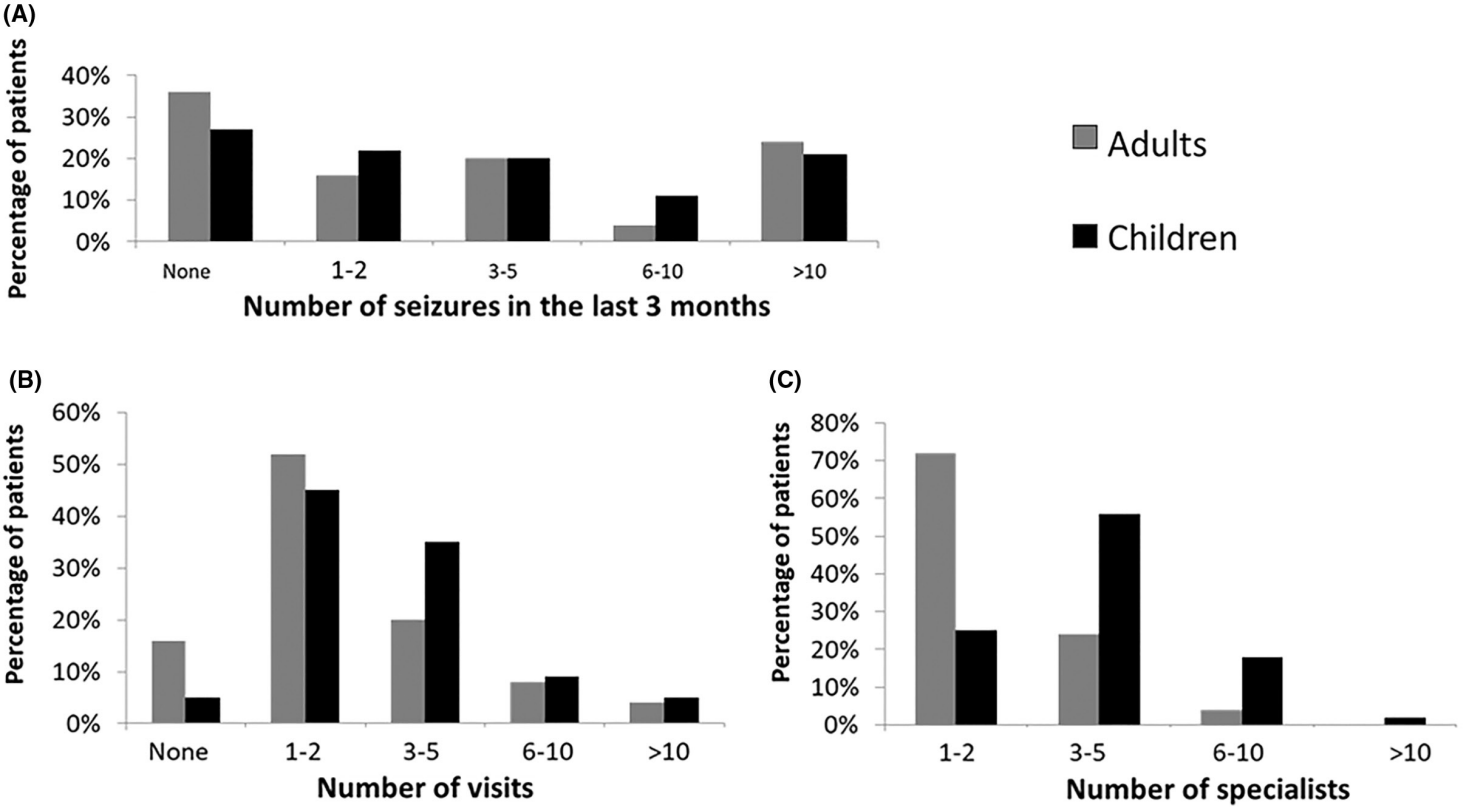
Healthcare services and seizure incidence among adult and pediatric patients. (A) Percentage of adults and children in relation to the number of seizures in the last 3 months. *p* = 0.2231. (B) Percentage of adults and children based on the number of hospital visits (excluding visits to pediatric neurologists) in the last year. *p* = 0.0325. (C) Percentage of adult and pediatric patients who visited different specialists, segmented by number of specialists visited (1–2, 3–5, 6–10, and >10) *p* < 0.0001.

One of the hallmarks of DS patient management relies in the transition from infant to adult healthcare.^20^ In our cohort, the number of seizures in the 3 months prior to the study was very similar between children and adults. However, a slightly higher percentage of adults were seizure‐free during this period (36%, compared to 27% of children, Figure 5A).

Seventy‐two percent (88/122) of the patients were referred to the PN by the emergency pediatrician, 19% (23/122) by their PCP, 7% (9/122) by the hospital pediatrician from outpatient clinics, and less than 2% (2/122) by their private pediatrician. The age of referral of the patient to a PN ranged from 0 to 36 months, with most of them (90%) being referred between 3 and 12 months.

When analyzing the number of visits to a secondary or tertiary healthcare specialist in the last year prior to the survey, the distribution between children and adults was slightly different. However, most patients in the study sample had to go to a specialist between 1 and 5 times in the year before the study (72% in children; 80% in adults). Interestingly, only 5% of children had not seen a specialist in the 12 months prior to the study compared to 16% of adults (Figure 5B). Almost half of the study participants (49%) visited between 3 and 5 specialists other than the PN, followed by 34% of patients who visited between 1 and 2 specialists. A small proportion (2%) had seen more than 10 specialists. Among the adult participants, the majority (72%) had consulted with 1–2 different specialists (Figure 5C). When asked how caregivers perceive the relationship between the different professionals who treat the patients with DS, 76% of our cohort (93/122) perceived it as fluid, compared to 24% (29/122) who did not see it that way.

## 4 DISCUSSION

In this paper, DS diagnostic delay, the availability of diagnostic resources, and the relationship between PC and PNS in the Spanish NHS were assessed. Results from two parallel surveys to PCPs and caregivers of DS patients indicate that there is still an unmet need to improve DS management and to expand education in DS among PCPs in Spain.

Previous studies suggest that the working conditions of PC professionals do not meet the minimum requirements that ensure proper clinical care, favoring professional burnout and stress.^21^, ^22^ In addition, the motivating aspects that could alleviate professional burnout are also very deficient. Training, teaching, and research are reviled, as the institutions do not facilitate their access or provide resources in PC.^23^

Our data show that there is a need for referral guidelines to PNs for suspected cases of DS and other complex epilepsies, unifying criteria while guaranteeing seizure and SE management in PC centers. It would be beneficial to compare these referral rates with those from other countries or regions to understand the differences in healthcare practices. Unfortunately, such specific comparative data may not be readily available in the literature. An improvement in early diagnosis of DS goes through the improvement of communication between PCPs and PNs, as well as with the rest of the patient care network.^16^, ^24^, ^25^, ^26^ The development of clear and consistent guidelines for the referral of patients from PC to the hospital PNs, with contributions from both PCPs, PNs, and PAGs seem essential.^16^, ^27^ In addition, the provision of material for the control of seizures and SE in PC centers is insufficient, according to the data. However, in our study, the percentage of patients referred by a PCP was much lower than in others.^28^ The lack of recommended treatments for the management of prolonged seizures available in PC centers hinders the effective treatment of seizures and increases its risk of evolution to SE, which implies a higher danger of complications, ICU admissions, and even death, especially in patients living in nuclei far from tertiary care hospitals.^16^

Initiatives will be undertaken to formulate guidelines through collaboration with PCPs, NPs, PAGs, and healthcare authorities to standardize referral criteria and the implementation of multidisciplinary groups for a holistic treatment of the disease. Future studies should focus on how these guidelines were received and applied and on measuring the effectiveness of these measures. Moreover, the outreach of an existing university summer course about the diagnosis, treatment, and management of DS will be enhanced through collaboration with professional societies, potentially elevating DS awareness among PCPs. However, the high workload of PCPs is recognized, and therefore concise educational materials that convey essential information should be preferred. Additionally, instructional resources for caregivers will be developed to empower families impacted by DS, enabling them to make informed decisions regarding the treatment they receive.

This study also sheds light on the diagnostic challenges faced by patients and their families. The diagnostic delay can be attributed to the rarity of DS and the similarity of its early symptoms to other conditions. In our study, 14% of families suggested the diagnosis to their doctors, a lower percentage than in a 2018 study by Lagae et al., where it was 24%.^17^ Our data on diagnostic delays and misdiagnosis (70%) align with prior research emphasizing the need for early diagnosis and awareness among HCPs.^29^ Delays in diagnosis often lead to the use of medications that are contraindicated for DS (especially sodium channel blockers), which has been related to an increased number of seizures^12^ and cognitive impairment,^30^ influencing disease prognosis.^18^

Our study coincides with others in the marked delay in the diagnosis of DS in adults.^28^, ^29^ This study shows that the diagnostic delay and the age at diagnosis are decreased in younger patients compared to older ones. The shorter diagnostic delay in young patients is encouraging and probably reflects the increased awareness of the disease and the availability of genetic testing.^30^ Given these findings and the importance of a correct diagnosis for disease management and prognosis,^31^ there is a critical need for specific efforts to identify undiagnosed Dravet syndrome patients in adulthood. For these reasons, projects are being initiated in partnership with daycare centers to enhance the diagnosis of DS in adults who may have been previously misdiagnosed. Furthermore, guidelines are being crafted to streamline the referral process from NPs to adult neurologists for adolescents. This effort involves collaboration with NPs, neurologists, PAGs, and social workers, aiming to ease the transition for patients and empower them in their healthcare journey.

Our data confirm that younger children attend more hospital consultations,^28^ while adults consume fewer resources. Nonetheless, more studies are needed to assess whether an inappropriate transition to adult care affects these data.

Despite the contributions this work provides to the field, there are some limitations that should be addressed. The survey of pediatricians may present a subjectivity bias; however, it provides an image that derives from the experience gained in the usual clinic. Furthermore, since the questionnaire was distributed via newsletters and social media, the response rate from PCPs is unknown. Furthermore, the responses obtained from caregivers may be biased not only by a lack of knowledge of seizure types or EEG results but also by the possible inaccuracy of their recollections. In addition, despite the efforts made to obtain responses from the entire Spanish territory, PCPs were predominantly located in Valencia, while caregivers were primarily residing in Madrid. This geographical distribution introduces a potential bias to the study results. Nevertheless, it is noteworthy that the significant number of caregivers in Madrid aligns with Spain's demographic distribution^32^ and the patients’ membership of the Spanish PAG. Another limitation of the questionnaire to PCPs is the focus on the referral timing and mode, the communication protocols between PCPs and NPs and between PCPs and PAGs, while lacking information about what is done after the referral.

While it is acknowledged that connecting patients with PAGs is beneficial following a diagnosis of a serious illness, many HCPs are not fully aware of the extensive resources available through these organizations, which can aid both patients and professionals. It appears that the Spanish NHS may have an opportunity for improvement to enhance PCPs education regarding the role of these groups.^17^ The Dravet Syndrome Foundation Spain has undertaken dissemination initiatives by distributing posters and flyers to primary care centers and major hospitals. These efforts aim to raise awareness about DS and the existence of a PAG specifically dedicated to DS in Spain. In addition, the active participation in scientific and medical conferences of both HCPs and PAGs fosters collaboration and spreads the knowledge of PAGs activities among medical societies.

Regarding caregivers, 100% of respondents belonged to a patient association. These persons were likely caregivers with high access to information and degree of commitment to both the patient and their condition. This exposure may represent a potential limitation of the study, given that such caregivers could exhibit bias in their responses, potentially differing from those of less informed caregivers.^15^

In summary, this study emphasizes the importance of improved vigilance in recognizing the challenges associated with DS. Increased education of HCPs, early diagnosis, implementation of improved treatment strategies, and fostering collaborative efforts among healthcare professionals are crucial for effectively managing this complex and rare disorder. Additionally, the pivotal role played by PAGs in raising awareness, offering support, and advancing research cannot be overstated. Moving forward, continued scientific studies and interdisciplinary research efforts may contribute to improve the primary care of DS patients.

## CONFLICT OF INTEREST STATEMENT

JAA is president of the Dravet Syndrome Foundation Spain (DSF). SG is the scientific director of Dravet Syndrome Foundation Spain (DSF). They and/or the DSF have received grants and/or financial support from GW Pharma, Zogenix, Ovid Therapeutics, Encoded Therapeutics, Biocodex, Praxis, Stoke, Takeda, UCB, Epygenix, Jazz Pharmaceuticals, and StrideBio to help carry out some of the DSF's foundational activities or provide consulting services. The honoraria have always been donated directly or indirectly to the DSF. ECM is currently an employee of Biocodex. She has no conflict of interests. We confirm that we have read the journal's position on issues involved in ethical publication and affirm that this report is consistent with those guidelines.

## Supporting information


Appendix S1.

## Data Availability

The data that support the findings of this study are available on request from the corresponding author. The data are not publicly available due to privacy or ethical restrictions.
