# Supplementary material for: Navigating Dravet syndrome in Spain: A cross‐sectional study of diagnosis, management, and care coordination
Source: Epilepsia Open. 2024 Jul 10;9(5):1806–15. doi: 10.1002/epi4.13012 (PMC11450586; doi:10.1002/epi4.13012)
Supplement: Supplementary file 1 — Appendix S1. [file EPI4-9-1806-s001.docx]

**SUPPORTING INFORMATION**

Table S1 Provinces of origin of the pediatricians interviewed.

| **Province** | **N** | **Percentage** |
| --- | --- | --- |
| Valencia | 20 | 32.8% |
| Madrid | 14 | 23.0% |
| Barcelona | 10 | 16.4% |
| Murcia | 4 | 6.6% |
| Navarra | 4 | 6.6% |
| Gipuzkoa | 4 | 6.6% |
| Toledo | 1 | 1.6% |
| Alicante | 1 | 1.6% |
| Castellón | 1 | 1.6% |
| Balears (Illes) | 1 | 1.6% |
| Lleida | 1 | 1.6% |

Table S2 Features of the cohort of the PCPs interviewed.

|  | **RANGE** | **MEAN** |
| --- | --- | --- |
| **AGE (YEARS)** | 33-66 | 50.2 |
| **YEARS OF EXPERIENCE IN PC** | 8- 39 | 23.4 |
| **PCPs PATIENT QUOTA** | 700-1220 | 974 |

Table S3 Provinces of origin of the parents interviewed.

| **Province** | **N** |
| --- | --- |
| Madrid | 27 |
| Valencia/València | 9 |
| Sevilla | 8 |
| Barcelona | 8 |
| Alicante/Alacant | 7 |
| Valladolid | 5 |
| Zaragoza | 5 |
| Cáceres | 4 |
| Cádiz | 4 |
| Málaga | 4 |
| Murcia | 4 |
| Rioja (La) | 3 |
| Asturias | 3 |
| Gipuzkoa | 3 |
| Jaén | 2 |
| Vizcaya/Bizkaia | 2 |
| Albacete | 2 |
| Pontevedra | 2 |
| Palmas (Las) | 2 |
| Balears (Illes) | 2 |
| Navarra | 2 |
| Lleida | 2 |
| Toledo | 1 |
| Granada | 1 |
| Melilla | 1 |
| León | 1 |
| Tarragona | 1 |
| Ciudad Real | 1 |
| Coruña (A) | 1 |
| Santa Cruz de Tenerife | 1 |
| Burgos | 1 |
| Córdoba | 1 |
| Castellón/Castelló | 1 |
| Soria | 1 |

Table S4 Sociodemographic data of caregivers and patients (N = 122).

| **CAREGIVER** | Mother | N=87 | 71% |
| --- | --- | --- | --- |
|  | Father | N=35 | 29% |
|  | Age (years) | 43.8 (range, 28-66) |  |
|  | Patient living with both parents | N=107 | 88% |
|  | Patient living with a single parent | N=15 | 12% |
| **PATIENT** | Male | N=66 | 54% |
|  | Female | N=54 | 46% |
|  | Age | 12.14 years (Range 8 months-40 years) |  |
|  | <2 years | N=10 | 8% |
|  | 2-7 years | N=36 | 30% |
|  | 8-17 years | N=51 | 42% |
|  | >18 years | N=20 | 20% |
|  | Patient living with siblings | N=87 | 71% |

Questionnaire to PCPs

**Socio-healthcare analysis and proposals for improvement of pediatric care of Dravet syndrome**

**Survey for Primary Care Pediatricians**

1. Are you (or have you been) a primary care pediatrician in Spain with more than two years of work experience?
   - Yes
   - No
2. Indicate your age in years
3. Indicate your years of experience in pediatrics (including residency)
4. Indicate your province of work
   - List of Spanish provinces
5. Indicate your patient quota
6. Indicate your reference children’s hospital
7. Indicate the treatments available at your workplace for treating a prolonged seizure or status epilepticus (multiple choice):
   - Diazepam
   - Midazolam
   - Age-appropriate intravenous access
   - Intraosseous
   - Pediatric nurse trained in vascular access
   - Intravenous Valproate
   - Intravenous Phenytoin
8. Do you refer all seizures to the reference hospital?
   - Yes
   - No
9. If the answer is no, indicate which ones you refer from the following (multiple choice)
   - Status epilepticus
   - Atypical crisis
   - Fever without a source
   - Association with some other neurological alteration
   - Others: free text
10. In your usual practice, in a child under one year of age with a febrile seizure, who has been treated by you or seen in the emergency department and sent home, when do you consider referral to a neuropediatric consultation?
    - Atypical crisis
    - Recurrent crisis
    - Association with some other neurological alteration
    - Others: free text
11. In your usual practice, do you request EEG or imaging tests in children with suspected complex crises, do you refer to neuropediatric consultation or both simultaneously?
    - I refer to NP consultation
    - I request EEG or imaging first
    - Both simultaneously
12. From your experience, how long is the average delay for a first Neuropediatric consultation at your reference hospital?
    - Less than 1 month
    - 1-3 months
    - 3-6 months
    - More than 6 months.
13. Do you think there is good communication between primary care and hospital services in your reference area?
    - Yes
    - No
14. Have you ever needed to contact the Neuropediatrician in your reference area to treat a case of one of your patients that you manage together?
    - Yes
    - No
15. If the answer is yes, how did you contact?
    - By phone
    - By email
    - Others: free text
16. Are there agreed protocols between Neuropediatrics and Primary Care in your area for the management of seizures and status epilepticus?
    - Yes
    - No
17. How do you think communication and coordination between primary care and hospital services could be improved, especially for the management of children with chronic, serious conditions that result in many visits to primary care, emergency departments, and various hospital services?
    - Free text
18. Do you know or have you ever contacted patient associations when a child in your care has been diagnosed with a rare, chronic disease or one with potential poor progression?
    - Yes
    - No
19. Do you usually recommend to your patients and families that they get to know the patient associations of the conditions they are diagnosed with?
    - Yes
    - No
20. In conditions whose diagnosis is genetic, are you aware of the possibility that the genetic test can be performed by some patient associations with a medical report at no cost?
    - Yes
    - No
21. Do you know genetic origin epilepsies?
    - Yes
    - No
22. Do you know Dravet syndrome?
    - Yes

Questionnaire to caregivers

**Socio-healthcare analysis and proposals for improvement of pediatric care of Dravet syndrome**

**Survey for Caregivers**

1. Are you of legal age and reside in Spain?
   - Yes
   - No
2. What is your province of residence?
   - List of Spanish provinces
3. What is your relationship to the person with Dravet syndrome?
   - Father
   - Mother
   - Other family member
   - Professional caregiver
4. How old are you (in years)?
5. How old is the person with Dravet syndrome (in years)?
6. What is the sexual identity of the person with Dravet syndrome?
   - Female
   - Male
7. What is the composition of family unit?
   - Caregiver and patient
   - Both parents and patient
   - Both parents, patient and sibling(s)
8. Do other children live with the patient?
   - Yes
   - No
9. At what age did the seizures begin? (in months)
10. What was the first type of seizure the patient had?
    - Generalized tonic-clonic seizures
    - Absence seizures
    - Myoclonic seizures
    - Tonic seizures
    - Atonic seizures
    - Focal seizures
    - Other: free text
11. The first seizure was related to (multiple choice):
    - Heat
    - Excitement
    - Fever
    - Infection
    - Immunization
    - None of the above
12. Was the first seizure febrile?
    - Yes
    - No
13. Approximately how many seizures did the patient have in the first year of life?
    - From 1 to 5
    - From 1 to 10
    - From 11 to 20
    - More than 20
    - None
14. How many status epilepticus did the patient have in the first year of life? (defined as seizures lasting more than 30 minutes, or shorter seizures recurring without recovery of consciousness between them)
15. What types of seizures did they have before being evaluated at the Neuropediatric clinic? (excluding occasional attention from the Neuropediatrician in Emergencies) (multiple choice)
    - Generalized tonic-clonic seizures
    - Absence seizures
    - Myoclonic seizures
    - Tonic seizures
    - Atonic seizures
    - Focal seizures
    - Other: free text
16. How old were they when they were referred to the specialized Neuropediatric Service? (in months)
17. Who referred you to the specialized Neuropediatric Service?
    - Emergency service
    - Primary care pediatrician
    - Other: free text
18. How much time passed between the first epileptic seizure and being referred to the specialized Neuropediatric Service?
    - Less than 1 month
    - 1-3 months
    - 3-6 moths
    - More than 6 months
19. How much time passed between being referred to the specialized Neuropediatric Service and the first consultation with the Neuropediatrician?
    - Less than 1 month
    - 1-3 months
    - 3-6 moths
    - More than 6 months
20. How old was the patient when they had their first electroencephalogram (EEG)?
    - Less than 1 year old
    - 1 year old
    - 2 years old
    - 3 years old
    - More than 3 years old
21. What was the result of the first EEG?
    - Normal
    - Abnormal/Pathological
    - I am not sure
22. Was a pathological EEG obtained at any time?
    - Yes
    - No
    - I am not sure
23. In case the response was yes, how old was the patient when they presented a pathological EEG?
    - Less than 1 year old
    - 1 year old
    - 2 years old
    - 3 years old
    - More than 3 years old
24. How old was the patient when you were given the clinical diagnosis of Dravet syndrome (in years)?
25. How many years passed between the first seizure and the clinical diagnosis of Dravet syndrome?
26. How many years passed between the first visit to the Neuropediatric clinic and the clinical diagnosis of Dravet syndrome?
27. Was a genetic test ever requested for your child?
    - Yes
    - No
28. At what age was the genetic test requested (in years)?
29. Were you initially given a different diagnosis than Dravet syndrome?
    - Yes
    - No
30. What was the initial diagnosis?
    - Free text
31. Did you or any family member suggest the diagnosis of Dravet to your doctors?
    - Yes
    - No
32. How many visits to the Emergency Service, for seizures, were needed before being diagnosed with Dravet syndrome?
    - From 1 to 5
    - From 6 to 10
    - From 11 to 20
    - More than 20
33. Did you receive information or did your doctors recommend knowing or belonging to any patient or support group at the time of diagnosis?
    - Yes
    - No
34. Do you belong to any Dravet syndrome, epilepsy, and/or support patient group?
    - Yes
    - No
35. How did you meet them (multiple choice)?
    - Internet search
    - Social networks
    - Other families
    - Doctor’s recommendation
    - Other: free text
36. Do you perceive fluid communication between the different specialists treating your child?
    - Yes
    - No
37. Have you ever tried to combine appointments on the same day to avoid trips to the hospital?
    - Yes
    - No
38. If you have tried, how did you perceive the treatment received? (free text)
39. How do you think coordination between the different levels of care and specialists attending your child could be improved, to improve the quality of life of your child and your family? (free text)
40. What antiseizure medication are you currently taking as part of your regular treatment? (multiple choice)
    - Eslicarbazepine acetate
    - Acetazolamide
    - Valproic acid (or sodium valproate)
    - Brivaracetam
    - Potassium bromide
    - Cannabidiol (Epidyolex)
    - Carbamazepine
    - Cenobamate
    - Clobazam (Frisium)
    - Clonazepam
    - Diazepam
    - Stiripentol (Diacomit)
    - Ethosuximide
    - Everolimus
    - Felbamate
    - Fenfluramine
    - Phenytoin
    - Phenobarbital
    - Gabapentin
    - Lacosamide
    - Lamotrigine
    - Levetiracetam
    - Midazolam (Buccolam)
    - Oxcarbazepine
    - Perampanel
    - Piracetam
    - Pregabalin
    - Primidone
    - Rufinamide
    - Tiagabine
    - Topiramate
    - Vigabatrin
    - Zonisamide
    - Other
41. What antiseizure medication have you taken in the past and are not currently taking as part of your regular treatment? (multiple choice)
    - Eslicarbazepine acetate
    - Acetazolamide
    - Valproic acid (or sodium valproate)
    - Brivaracetam
    - Potassium bromide
    - Cannabidiol (Epidyolex)
    - Carbamazepine
    - Cenobamate
    - Clobazam (Frisium)
    - Clonazepam
    - Diazepam
    - Stiripentol (Diacomit)
    - Ethosuximide
    - Everolimus
    - Felbamate
    - Fenfluramine
    - Phenytoin
    - Phenobarbital
    - Gabapentin
    - Lacosamide
    - Lamotrigine
    - Levetiracetam
    - Midazolam (Buccolam)
    - Oxcarbazepine
    - Perampanel
    - Piracetam
    - Pregabalin
    - Primidone
    - Rufinamide
    - Tiagabine
    - Topiramate
    - Vigabatrin
    - Zonisamide
    - Other
42. At any point, was the patient treated with antiseizure medication that are sodium channel blockers?
    - No, never
    - Lamotrigine (Lamictal)
    - Phenytoin (Dilantin, Epanutin)
    - Lacosamide (Vimpat)
    - Fosphenytoin (Cerebyx, Prodilantin)
    - Carbamazepine (Tegretol, Carbatrol, Epitol, Finlepsin, Sirtal, Stazepine)
    - Oxcarbazepine (Trileptal)
    - Rufinamide (Inovelon)
    - Tiagabine (Gabitril)
    - Vigabatrin (Sabril, Sabrilan, Sabrilex)
43. Is the person with Dravet syndrome also on any of the following treatments? (multiple choice)
    - Vitamin or amino acid supplements
    - Ketogenic diet
    - Vagus nerve stimulation
    - ADHD treatment
    - Antipsychotics
    - Nutritional treatment
    - None of the above
44. In the case of prolonged seizures, what is the usual rescue medication used?
    - Diazepam (Stesolid)
    - Midazolam (Buccolam)
    - Other
    - None
45. Does your child have an updated emergency protocol for prolonged crises that they always carry with them so that there is a consensus in any Emergency Service?
    - Yes
    - No
46. How many seizures has the person with Dravet syndrome had in the last 3 months?

- None
- From 1 to 2
- From 3 to 5
- From 6 to 10
- More than 10

1. Excluding visits to the Emergency Service, how many times have they gone to Neuropediatrics or other related specialists in the last 12 months?

- None
- From 1 to 2
- From 3 to 5
- From 6 to 10
- More than 10

1. How many different specialists do you visit with your child? Among these are the neuropediatrician, rehabilitator, psychologist, ENT, cardiologist, others.

- None
- From 1 to 2
- From 3 to 5
- From 6 to 10
- More than 10
